# An Observational Study of Hypertension and Thromboembolism Among Transgender Patients Using Gender-Affirming Hormone Therapy

**DOI:** 10.1089/trgh.2019.0061

**Published:** 2020-03-16

**Authors:** Maria Pyra, Isabel Casimiro, Laura Rusie, Nat Ross, Cori Blum, Kristin Keglovitz Baker, Andie Baker, John Schneider

**Affiliations:** ^1^ Howard Brown Health Center, Chicago, Illinois.; ^2^ Chicago Center for HIV Elimination, University of Chicago, Chicago, Illinois.; ^3^ Section of Adult and Pediatric Endocrinology, Diabetes, and Metabolism, University of Chicago Medicine, Chicago, Illinois.

**Keywords:** hypertension, sex hormones, thromboembolism

## Abstract

**Purpose:** Given evidence from cisgender patients that sex hormones can impact risk for some forms of cardiovascular disease (CVD), there are concerns regarding CVD among transgender patients using gender-affirming hormone therapy (HT).

**Methods:** Using a retrospective cohort at a U.S. urban federally qualified health center (FQHC) focused on sexual and gender minority health, we examined associations between HT in transgender patients and two specific CVD outcomes, hypertension (HTN) and thromboembolism (TE). We assessed outcomes by ICD-10 codes in electronic medical records (EMR) of 4402 transgender patients. Hormone use was assessed both by blood concentrations and by prescriptions, from EMR.

**Results:** Nineteen transwomen (TW) (0.8%) had a TE and 49 (2.1%) developed HTN; among transmen (TM), 27 (1.5%) developed HTN and there were no significant associations between hormones and HTN. Among transwomen, there was no association between TE and HT as assessed by blood concentrations. However, recent progestin prescriptions were associated with an increased odds of TE (adjusted odds ratio [aOR] 2.95 [95% confidence interval; CI 1.02–8.57]), with possibly differential effects for medroxyprogesterone acetate versus micronized progesterone. Higher total testosterone blood concentrations were associated with greater odds of HTN in TW (aOR 1.16 [95% CI 1.01–1.33]), after controlling for body mass index. Among TW, ever having a progestin prescription was protective for HTN (aOR 0.36 [95% CI 0.15–0.87]).

**Conclusion:** We found no associations between HT and HTN among TM, More research is needed to examine the effect of recent progestin, specifically medroxyprogesterone acetate, on TE among transwomen. The protective association between progestins and HTN among TW is reassuring.

## Introduction

The goal of gender affirming hormone therapy (HT) is to suppress endogenous sex hormones and maintain sex hormone levels within the recommended range for the person's affirmed gender.^1,2^ Most of the research regarding the relationship between the use of sex hormones and cardiovascular disease (CVD) has been obtained from cisgender populations (those whose gender identity aligns with their sex at birth).^3–5^ However, there are important differences in hormonal formulations, dosing, therapy duration, endogenous hormone exposure, and comorbidities between cisgender and transgender patients.^6^ There is limited research regarding the long-term safety of gender-affirming hormonal therapy for transgender patients, with most of the studies coming primarily from Europe.^6–10^ Given differences in health care access and preferred regimens, additional information is clearly needed regarding HT and CVD risk among transgender patients in the United States.^11,12^

Feminizing HT consists of an estrogen formulation that is usually co-administered with an androgen antagonist (such as spironolactone, commonly used in the United States, or cyproterone acetate, CPA, an antiandrogen agent with progestational properties that is commonly used in Europe). In some cases finasteride is used to inhibit the effects of endogenous dihydrotestosterone; while some patients may also add-back testosterone to maintain energy and libido. Data from cisgender women have suggested that exogenous estrogen and/or progesterone is associated with an increased risk of thromboembolic events in trans women (TW).^13^ However, it is thought that transdermal and parenteral routes of administration have less of a thromboembolic risk since they avoid the first pass effect.^14–16^ For masculinizing therapy, testosterone is used as HT in trans men (TM), in various formulations such as parenteral, topical, or transdermal; patients may also use topical estrogens to counteract vaginal atrophy.

Sex hormones, including testosterone, estrogens, and progestins, can affect CVD risk in several ways. Testosterone therapy, when given to either TM or hypogonadal cismen, can result in erythrocytosis, lipid changes, and increased blood pressure.^17,18^ Estrogens can shift the hemostatic system toward thrombosis and have been shown to be associated with increased thrombotic risk in postmenopausal cis women.^13^ In TW, oral ethinyl estradiol has been associated with an overt increase in venous thromboembolism (TE), thus this particular formulation is no longer recommended.^19^ The effect of the currently widely prescribed HT on cardiac disease and mortality are not entirely clear and in some cases may be modified by aging.^5,11,20–23^

Therefore, our aim was to describe the rate of CVD among transgender patients using gender affirming HT in a large community health center that serves the largest transgender population in Illinois. Furthermore, we explored associations between each category of hormone and specific CVD outcomes, stratified by affirmed gender. We also hypothesized that different routes of drug administration, particularly for estrogens, and formulations, specifically of progestins, may have different effects on CVD.

## Methods

### Data

We used electronic medical records (EMR) from an urban federally qualified health center (FQHC) that specializes in sexual and gender minority health (Howard Brown Health, Chicago, IL). In 2018, more than 35,000 patients from over 2000 zip codes were seen for medical care. We initially extracted data on all living transgender patients from October 2006 to October 2018; while gender is self-reported, we also used an algorithm to determine gender if there is conflicting information. Only 76 out of the 3529 total transwomen and 37 of the 2983 total transmen seen at the health center did not have a history of hormone use; however, nonusers were much more likely to have CVD events (30% of transwomen and 43% of transmen). This suggests that poor health may have precluded them from receiving HT, though patients may also have chosen not to use hormones. Therefore, our analysis included all transgender patients ages 20–70 with a history of hormone use (which varies by type of hormone and duration of use) and we excluded those with less than 6 months since their first hormone prescription.

HT use was assessed by concentrations of estradiol and testosterone, as ordered by the clinician for regular monitoring, and prescription records for HT. Lab values were from most recent visit before CVD (but no more than 1 year prior) or before October 2018 for those without CVD; if multiple values were found from same visit date, the average was used. Values less than the lower limit were set to half the lower limit.

We defined CVD using ICD-10 codes for TE (I26, I65, I66, I80–I82, I87), hypertension (HTN) (I10), coronary artery disease (I21, I25.1–125.6, I63, I67), and hyperlipidemia (E78). All cases were chart reviewed and verified by a physician. There were no cases of incident hyperlipidemia in our sample (which may have been due to infrequent testing), 13 cases of coronary artery disease (11 in transwomen and 2 in transmen), and 3 cases of incident TE in transmen. Therefore, we focused our analysis on incident TE in transwomen and incident HTN in both transwomen and transmen.

### Statistical analysis

We modeled TE or HTN events as a binary outcome with concentrations of estradiol and total testosterone from patients' lab results as predictors, using logistic regression and stratified by affirmed gender. The resulting odds ratios (ORs) are presented per 100 U change in concentration, to provide a meaningful context. Next, we adjusted for possible confounders, including age, race, insurance type, HIV status, sexual orientation, and time since first HT prescription; confounders that did not change the estimates and were not of independent interest were dropped. Finally, in the mediated model, we also tested body mass index (BMI), diabetes, cholesterol, and smoking status, as these may have been influenced by hormone use; items that did not change the estimates or had too much missing data were dropped from the model. We then repeated this model using the categorical versions of estradiol and total testosterone.

Next, we used recent prescription data (<1 year before TE or HTN event or before October 31, 2018) as the predictors and used similar adjusted and mediated logistic models. We then examined the route of estrogen and specific formulations of progestin. Finally, we used ever having a prescription for each hormone-route combination as the predictor, adjusting as described above. Additionally, for a subset of transwomen with reliable data, we determined the number of months of use for each hormone-route combination and modeled both HTN and TE with duration of use as the predictor.

For all analyses, when possible we stratified by age (20–44 vs. 45–70), BMI (under/healthy vs. overweight/obese/morbidly obese), and HIV status; due to small numbers, we were not able to test for interaction terms. All analyses were conducted with SAS 9.4 (SAS Institute).

## Results

Data from 2509 transwomen and 1893 transmen were used in these analyses (Table 1). The median age was 30 for transwomen, and 26 for transmen; both groups were mostly normal/overweight by BMI. The sample was predominantly White and had private insurance. HIV was more common among transwomen compared to transmen (13.4% vs. 0.7%). Median time since first hormone prescription was 2.6 years for transwomen and 2.2 years for transmen. Almost all transwomen ever had a prescription for estrogen (99%) or an androgen antagonist (AA)/finasteride (94.2%) at the clinic, while 29.8% had used a progestin; almost all transmen (99.6%) ever had a prescription for testosterone at the clinic.

**Table 1.** Patient Characteristics at Most Recent Visit

	Transgender women % (N)	Transgender men % (N)
*N*	2509	1893
Median age (range)	30 (20, 70)	26 (20, 67)
Median BMI (kg/m^2^) (range)	26.4 (12.9, 66)	27.6 (14.2, 67.9)
Underweight (≤18.5)	4.0% (99)	1.6% (31)
Normal (18.6–25)	37.3% (936)	31.8% (602)
Overweight (25.1–30)	27.0% (678)	27.7% (525)
Obese (30.1–40)	24.7% (620)	28.1% (533)
Class 3 Obese (≥40)	7.0% (176)	10.7% (203)
Sexual orientation		
Gay/Lesbian	24.4% (611)	12.3% (233)
Bisexual	20.1% (503)	10.6% (200)
Queer	12.8% (320)	34.3% (650)
Straight	20.3% (509)	23.8% (450)
Other	22.6% (566)	19.0% (360)
Race		
White	54.9% (1377)	66.0% (1250)
Black	18.6% (466)	12.5% (236)
Latinx	17.9% (448)	13.5% (256)
Other	8.7% (218)	8.0% (151)
Insurance status		
Private	42.7% (1072)	58.5% (1107)
Medicare	5.4% (135)	2.0% (37)
Medicaid	26.2% (656)	16.7% (316)
Sliding scale	25.8% (646)	22.9% (433)
HIV	13.4% (336)	0.7% (13)
Diabetes	3.3% (83)	3.0% (56)
Median year since first hormone prescription (range)	2.6 (0.5, 12.0)	2.2 (0.5, 11.9)
Ever smoker (missing=747)	38.9% (804)	37.9% (602)
Mean total cholesterol mg/dL (SD) (missing=378)	173.3 (36.3)	173.2 (37.0)
Ever use estrogen	99.0% (2485)	8.0% (152)
Ever use AA	94.2% (1364)	4.6% (87)
Ever use progestin	29.8% (748)	2.8% (53)
Ever use testosterone	1.6% (41)	99.6% (1886)

AA, androgen antagonist; BMI, body mass index; SD, standard deviation.

Among transwomen exposed to hormone treatment, 0.8% (19) had a TE, and 2.1% (49) developed HTN. Among transmen exposed to hormone treatment, 0.2% (3) developed TE, and 1.5% (28) developed HTN. Given the very small number of TE in transmen, association analyses were not done on TM as they would have been noninformative.

### Transmen and HTN

Among the 1363 transmen there were no significant associations between blood hormone levels and HTN diagnosis (Table 2). Among transmen, 96% had a recent testosterone prescription regardless of HTN diagnosis, while 3.7% of those with HTN and 2.3% of those without had a recent estrogen prescription, including for atrophic vaginitis, Pap smear preparation, and pelvic pain. There were no significant associations between HT prescriptions and HTN.

**Table 2.** Hormones Concentrations (Continuous and Categorical) and Odds of Thromboembolism and Hypertension

	TE (n=15)	No TE (n=2014)	Unadjusted OR^*^	Mediated OR^***^	HTN (n=43)	No HTN (n=1863)	Unadjusted OR^*^	Mediated OR^***^
Transgender women								
Mean estradiol^a^ (SD)	267.8 (305.0)	284.4 (334.1)	0.96 (0.80, 1.15)	0.99 (0.84, 1.18)	250.5 (358.9)	286.5 (336.9)	0.97 (0.88, 1.08)	1.02 (0.92, 1.13)
0–99 pg/mL	33.3% (5)	33.7% (679)	1.86 (0.53, 6.50)	1.34 (0.36, 4.91)	41.9% (18)	33.7% (629)	1.32 (0.60, 2.93)	0.89 (0.38, 2.08)
100–200 pg/mL	20.0% (3)	22.0% (443)	1.01 (0.26, 3.93)	0.93 (0.23, 3.69)	18.6% (8)	21.6% (403)	0.96 (0.41, 2.25)	0.98 (0.40, 2.43)
≥201 pg/mL	46.7% (7)	44.3% (892)	Ref	Ref	39.5% (17)	44.6% (831)	Ref	Ref
Mean total testosterone^a^ (SD)	91.8 (235.7)	136.7 (233.2)	0.88 (0.65, 1.19)	0.90 (0.65, 1.25)	166.2 (250.9)	137.4 (233.9)	1.04 (0.92, 1.17)	1.16 (1.01, 1.33)^#^
<50 ng/dL (ref ≥50)	86.7% (13)	66.2% (1334)	4.62 (0.91, 23.5)	4.24 (0.82, 21.9)	60.5% (26)	66.2% (1234)	0.91 (0.43, 1.90)	0.68 (0.31, 1.49)

	HTN (n=17)	No HTN (n=1347)	Unadjusted OR^*^	Mediated OR^***^				
Transgender men								
Mean estradiol^a^ (SD)	63.0 (24.9)	63.7 (74.3)	0.99 (0.50, 1.97)	0.85 (0.29, 2.46)				
<20 pg/mL (ref ≥20)	5.9% (1)	2.7% (36)	2.32 (0.30, 18.18)	2.99 (0.33, 27.40)				
Mean total testosterone^a^ (SD)	579.3 (311.0)	573.2 (350.8)	1.00 (0.88, 1.15)	1.05 (0.90, 1.21)				
0–399 ng/dL	23.5% (4)	31.9% (429)	0.81 (0.21, 3.06)	0.59 (0.14, 2.51)				
400–700 ng/dL	47.1% (8)	34.7% (467)	1.51 (0.49, 4.66)	1.60 (0.49, 5.30)				
>700 ng/dL	29.4% (5)	33.5% (451)	Ref	Ref				

^a^ Model results for continuous hormone levels are presented per 100-U change in concentration.

^*^ All hormones are included together in the unadjusted model. ^***^Includes race, insurance, HIV status, age, and BMI. ^#^*p*<0.05.

BMI, body mass index; HTN, hypertension; OR, odds ratio; TE, thromboembolism.

### Transwomen and TE

Of the 2029 transwomen with hormone concentrations available before a TE diagnosis, 20.0% of those with a TE and 22.0% of those without had measured estradiol levels within the target range of 100–200 pg/mL (Table 2); 86.7% of those with a TE and 6.26% of those without had levels in the target testosterone range of <50 ng/dL. There were no associations between TE and either estradiol or testosterone in any of the models. Progestin levels are not measured routinely in the course of gender-affirming care.

Among transwomen with TE, 84.0% had a recent (within prior year) estradiol prescription in any formulation (compared to 78.2% of those without TE); 57.9% had a recent AA prescription (compared to 61.0% without TE); and 26.3% had a recent progestin prescription (compared to 14.0% without TE) (Tables 3 and 4). There were no significant associations between TE and use of estradiol or an anti-androgen (Model 1, Table 5). However, recent progestin prescription was significantly associated with increased odds of TE in transwomen; after adjusting for race, insurance, HIV status, age, and BMI in the mediated model, the adjusted OR (aOR) was 2.95 (95% confidence interval [CI] 1.02–8.57, *p*=0.04) (Model 1, Table 5). Next, we used the same models, including specific routes of estrogen and formulations of progestin. The odds of HTN for medroxyprogesterone acetate (MPA) prescription appeared higher than the association between HTN and micronized progesterone (Model 2, Table 5). However, the CIs were very wide and the sample size is too small to be conclusive, as TE was a rare event, occurring in 1.4% (5) patients with a recent progesterone prescription and 4.6% (1) patient with a recent MPA prescription.

**Table 3.** Recent Hormone Prescriptions Among Transwomen, by Thromboembolism and Hypertension

	TE (n*=19), % (*n)	No TE (n*=2490), % (*n)	HTN (n*=49), % (*n)	No HTN (n*=2290), % (*n)
Estrogen	84 (16)	78.2 (1948)	73.5 (36)	78.7 (1801)
Estrogen-Oral	32 (6)	37.8 (940)	24.5 (12)	38.1 (872)
Estrogen-Patch	5.3 (1)	2.9 (73)	4.1 (2)	2.6 (59)
Estrogen-Gel	0 (0)	0.2 (5)	0 (0)	0.2 (4)
Estrogen-Injectable	52.6 (10)	38.6 (961)	44.9 (22)	39.1 (896)
Estrogen-Implant	0 (0)	0.2 (4)	0 (0)	0.2 (4)
Estrogen-Vaginal	0 (0)	0.1 (2)	0 (0)	0.1 (2)
AA/Finasteride	57.9 (11)	61.0 (1519)	51.0 (25)	61.5 (1408)
Progestin	26.3 (5)	14.0 (349)	8.2 (4)	14.5 (331)
Progestin-Oral	26.3 (5)	13.9 (345)	8.2 (4)	14.3 (327)
Progestin-Injection	0 (0)	0.2 (5)	0 (0)	0.2 (5)
Testosterone	0 (0)	0.7 (18)	2.0 (1)	0.8 (18)
Testosterone-Injection	—	—	2.0 (1)	0.4 (9)
Testosterone-Patch	—	—	—	—
Testosterone-Gel	—	—	0 (0)	0.4 (9)

**Table 4.** Details of Thromboembolism Cases Among Transwomen (*n*=22)

Case	Event and ICD-10 code	Age	HT formulation and duration	BMI	PMH/FH or known risks
1	DVT, provoked (I82.90)	47	Estradiol valerate IM 10–14 mg Q14 days×3 years	32.6	PMH: • Knee surgery 3 weeks
2	DVT (I82.89)	60	Estradiol valerate IM 40 mg Q14 days×3 years	29.7	No significant PMH or FH
3	DVT, provoked (I82.402)	25	Estradiol PO 6 mg Qd×2 years followed by: Estradiol valerate IM 20 mg Q14 days×2 years	41.2	PMH: • Immobilized×5 weeks before event due to foot injury • Fatty liver disease FH: Mother and Father: HLD, HTN
4	Thrombophlebitis (I80.9)	38	Estradiol 6 mg PO Qd×1 year, Micronized progesterone 100 mg×7 months, 4 years before event followed by: Estradiol valerate 20 mg IM Q14d and×5 years	27.5	PMH: • Smoker • Bipolar disease on lamictal and risperidone
5	Thrombophlebitis and PE, provoked (I26.99)	58	Estradiol valerate IM 20 mg Q14d×2 years and androgel 12.5 mg every other day	30.5	PMH: • HTN • Abdominal surgery 4 weeks prior • Thrombophlebitis at PICC line site
6	DVT, provoked (I82.40)	45	Estradiol valerate IM 8–20 mg Q7 days×4 years	34.5	PMH: • Surgery before TE • Schizoaffective d/o on ziprasidone FH: Multiple family members: HTN and heart disease
7	Superficial phlebitis (180.9)	23	Estradiol 4–6 mg PO Qd×4 months, MPA 2.5 mg Qd×2 months	28.6	PMH: • Schizophrenia on Abilify • 1 Day prior injected heroin into affected vein
9	PE (I26.99)	55	0.6 mg/24 h TD patches weekly×1 year followed by: Estradiol TD 0.2 mg/24 h patch Q 3d and premarin	28.3	PMH: • Schizoaffective disorder on Haldol, clozapine and divalproex
10	DVT (I82.4Z9)	50	Estradiol valerate IM 20 mg Q7 days×16 years	32.2	PMH: • Hx of DVT
12	Arterial micro emboli (MCA) (I66.09)	46	Estradiol 4–8 mg PO Qd×7 years	40.8	No significant PMH or FH
13	Superficial phlebitis (I80.9)	38	Estradiol valerate IM 20 mg Q14 days and premaren 5 mg Qd×5 years	46.6	PMH: • IV infusion placed in affected vein 1 week prior
14	PE, provoked (I26.99)	38	Conjugated estrogens 10 mg Qd×4 years followed by: Estradiol PO 6 mg Qd×3 years and MPA 2.5 mg Qd×1 year	28.4	PMH: • Ankle surgery 1 month prior
15	Portal vein thrombosis (I81)	36	Estradiol valerate IM 10–20 mg Q 14d×10 years	29.9	PMH: • HIV • Seizure disorder on lamictal • Asthma on prednisone
17	DVT and PE, provoked (I82.90, I26.99)	37	Premarin 25 mg Qd×3 months; Progesterone 100 mg Qd×2 months 1 year before incident followed by: Estradiol valerate IM 20 mg Q14 days×7 years	42.2	PMH: • HIV • Flight 1 week before DVT
18	Bilateral PEs, DVT (I82.90, I26.99)	72	Estradiol valerate 4–12 mg IM Q7 days×7 years	32.9	PMH: • Underlying malignancy diagnosed after incident (colon cancer)
19	Acute DVT, muscle spasm (I82.4Z)	29	Estradiol valerate IM Q14d×3 years, Progesterone 100 mg Qd×6 months	35.1	PMH: • Smoker (1 ppd) • Depression on Geodon
20	Acute DVT of legs, I82.40	51	Estradiol valerate IM 8 mg Q7d×2 months	48.1	PMH: • Schizophrenia on risperidone • Former smoker, HTN
21	Inferior vena cava syndrome (187.1)	24	Estradiol TD 0.1 mg/24 h twice a week×1 year Progesterone 100 mg Qd×10 months	25.8	No significant PMH or FH
22	Right leg pain, DVT (I82.4)	34	Estradiol valerate IM 40 mg Q14 days×10 years	26.6	FH: Mother HTN

FH, family history; DVI, deep vein thrombosis; PMH, patient medical history.

**Table 5.** Odds of Thromboembolism and Hypertension Among Transwomen, by Hormone Prescriptions

TE, Model 1	Recent prescriptions			History of prescriptions		
	Unadjusted^*^	Adjusted^**^	Mediated^***^	Unadjusted^*^	Adjusted^**^	Mediated^***^
Estrogen	1.52 (0.44, 5.28), *p*=0.51	1.54(0.43, 5.47), *p*=0.50	1.57 (0.44, 5.59), *p*=0.49	—	—	—
AA/finasteride	0.94 (0.37, 2.35), *p*=0.88	1.25 (0.48, 3.26), *p*=0.65	1.21 (0.46, 3.17), *p*=0.70	1.07 (0.14, 8.13), *p*=0.94	—	—
Progestin	2.23 (0.80, 6.24), *p*=0.12	2.98 (1.03, 8.63), *p*=0.04	2.95 (1.02, 8.57), *p*=0.04	1.07 (0.41, 2.84), *p*=0.88	—	—
TE, Model 2						
Estrogen-Oral	1.37 (0.38, 5.01), *p*=0.63	1.49 (0.41, 5.39), *p*=0.54	1.46 (0.40, 5.26), *p*=0.56	1.10 (0.39, 3.14), *p*=0.85	1.13 (0.39, 3.25), *p*=0.82	1.24 (0.42, 3.61), *p*=0.69
Estrogen-Patch	3.07 (0.34, 28.0), *p*=0.32	2.04 (0.22, 19.1), *p*=0.53	1.71 (0.18, 16.5), *p*=0.64	1.40 (0.46, 4.26), *p*=0.55	1.01 (0.32, 3.18), *p*=0.98	0.95 (0.30, 3.01), *p*=0.92
Estrogen-Injectable	2.35 (0.68, 8.16), *p*=0.17	2.21 (0.62, 7.86), *p*=0.21	2.25 (0.65, 7.83), *p*=0.20	2.65 (0.75, 9.36), *p*=0.13	2.26 (0.61, 8.34), *p*=0.22	2.21 (0.60, 8.22), *p*=0.23
AA/finasteride	1.04 (0.41, 2.65), *p*=0.93	1.37 (0.52, 3.62), *p*=0.52	1.32 (0.50, 3.50), *p*=0.57	1.12 (0.15, 8.49), *p*=0.91	1.91 (0.24, 15.2), *p*=0.54	1.99 (0.24, 16.3), *p*=0.52
Progesterone	1.73 (0.57, 5.29), *p*=0.33	2.28 (0.72, 7.21), *p*=0.16	2.27 (0.72, 7.16), *p*=0.16	0.80 (0.28, 2.25), *p*=0.66	0.99 (0.34, 2.87), *p*=0.97	1.00 (0.34, 2.94), *p*=0.99
MPA	11.11 (1.35, 91.4), *p*=0.02	17.7 (1.97, 159.6), *p*=0.01	20.0 (2.14, 187.2), *p*=0.009	1.83 (0.24, 14.0), *p*=0.56	1.44 (0.18, 11.5), *p*=0.73	1.61 (0.20, 13.1), *p*=0.65
HTN, Model 1						
Estrogen	0.60 (0.29, 1.23), *p*=0.16	0.66 (0.31, 1.42), *p*=0.29	0.67 (0.31, 1.43), *p*=0.29	0.76 (0.09, 6.47), *p*=0.79	0.32 (0.04, 2.79), *p*=0.29	0.39 (0.04, 3.90), *p*=0.42
AA/finasteride	0.56 (0.30, 1.06), *p*=0.07	0.88 (0.45, 1.74), *p*=0.72	0.93 (0.47, 1.85), *p*=0.83	0.51 (0.21, 1.27), *p*=0.14	1.25 (0.48, 3.27), *p*=0.64	1.38 (0.52, 3.70), *p*=0.51
Progestin	0.47 (0.17, 1.35), *p*=0.16	0.59 (0.20, 1.74), *p*=0.34	0.56 (0.19, 1.67), *p*=0.30	0.32 (0.14, 0.77), *p*=0.01	0.35 (0.15, 0.85), *p*=0.02	0.36 (0.15, 0.87), *p*=0.02
Testosterone	1.37 (0.16, 11.6), *p*=0.76	2.30 (0.26, 20.3), *p*=0.46	1.86 (0.21, 16.6), *p*=0.57	2.00 (0.41, 9.85), *p*=0.39	2.05 (0.40, 10.4), *p*=0.38	2.06 (0.39, 10.8), *p*=0.39
HTN, Model 2						
Estrogen-Oral	0.44 (0.20, 0.99), *p*=0.04	0.49 (0.21, 1.14), *p*=0.09	0.52 (0.22, 1.20), *p*=0.12	0.61 (0.32, 1.14), *p*=0.12	0.59 (0.30, 1.16), *p*=0.12	0.68 (0.34, 1.35), *p*=0.26
Estrogen-Patch	1.09 (0.24, 5.00), *p*=0.91	0.55 (0.11, 2.72), *p*=0.46	0.38 (0.06, 2.26), *p*=0.28	2.01 (1.03, 3.93), *p*=0.04	1.17 (0.57, 2.41), *p*=0.66	1.18 (0.56, 2.49), *p*=0.66
Estrogen-Injectable	0.76 (0.36, 1.60), *p*=0.47	0.89 (0.40, 1.99), *p*=0.77	0.86 (0.39, 1.92), *p*=0.71	0.77 (0.42, 1.44), *p*=0.41	0.70 (0.35, 1.39), *p*=0.31	0.72 (0.35, 1.45), *p*=0.35
AA/finasteride	0.61 (0.33, 1.14), *p*=0.12	0.96 (0.49, 1.88), *p*=0.89	1.01 (0.51, 2.01), *p*=0.97	0.53 (0.21, 1.31), *p*=0.16	1.28 (0.49, 3.33), *p*=0.61	1.43 (0.53, 3.86), *p*=0.47
Progesterone	0.50 (0.18, 1.43), *p*=0.19	0.60 (0.20, 1.75), *p*=0.34	0.58 (0.19, 1.72), *p*=0.32	0.36 (0.15, 0.87), *p*=0.02	0.45 (0.18, 1.11), *p*=0.08	0.46 (0.18, 1.13), *p*=0.08
MPA	—	—	—	0.75 (0.10, 5.58), *p*=0.77	0.39 (0.05, 3.20), *p*=0.38	0.39 (0.05, 3.37), *p*=0.39
Testosterone	1.53 (0.18, 12.8), *p*=0.69	2.86 (0.32, 25.5), *p*=0.34	2.13 (0.24, 19.3), *p*=0.50	2.00 (0.44, 9.16), *p*=0.37	2.49 (0.52, 12.0), *p*=0.25	2.43 (0.51, 11.6), *p*=0.26

^*^ All hormones are included together in the unadjusted model.

^**^ Includes race, insurance, HIV status, and age.

^***^ Also includes BMI.

MPA, medroxyprogesterone acetate.

When modeling ever having an HT prescription, there was no association with TE and progestin (nor any other hormone) (Table 5).

### Transwomen and HTN

There were 49 transwomen with HTN. Among those aged 18–39, 0.7% had incident HTN, age 40–59, 7.0%, and age 60 and older, 10.5%, compared to national prevalence rates of 7.5%, 33.2%, and 63.1% by age, respectively.^24^ There were 1907 transwomen with a hormone concentration available before an HTN diagnosis; there were no associations between estradiol levels and HTN (Table 2). However, in the mediated model adjusted for BMI, increased levels of endogenous testosterone were associated with an increased odds of HTN; for every 100 U increase in testosterone, there was a 16% increased odds of HTN for transwomen that was statistically significant (*p*=0.04, Table 2).

We stratified the mediated associations between hormone concentrations and HTN by age for transwomen. Among younger transwomen (20–44), estradiol appeared protective but this was not significant (aOR 0.81 [0.58, 1.12], *p*=0.20) and there was no association between HTN and testosterone levels (*p*=0.82). Among older transwomen (45–70), estradiol appeared to increase odds of HTN but this was not significant (aOR 1.11 [95% CI 0.98–1.25], *p*=0.09). However, the odds of increased HTN and increased endogenous testosterone levels was almost significant in older transwomen (aOR 1.21 [95% CI 1.00–1.46], *p*=0.05), while there was no association among younger women (aOR 1.03 [95% CI 0.80–1.32], *p*=0.82). We also examined the effects of testosterone by AA or finasteride use. For recent finasteride users, there was no significant association between testosterone and HTN (aOR 0.78 [95% CI 0.33–1.81], *p*=0.56); however among nonusers, the OR was 1.19 (95% CI 1.04–1.37, *p*=0.01) after adjusting for age and BMI. There were no associations for either spironolactone users or nonusers.

Among transwomen with HTN, 73.5% had a recent estrogen prescription (compared to 78.7% of those without HTN); 51.0% had a recent AA/finasteride prescription (compared to 61.5% without HTN); 8.2% had a recent progestin prescription (compared to 14.5% without HTN); and 2% had a recent testosterone prescription (compared to 0.8% of those without HTN) (Table 3). There was no association between recent hormone prescriptions and HTN (Model 1, Table 5). Transwomen who ever had a progestin prescription had lower odds of HTN (aOR 0.36 [95% CI 0.15–0.87]), with no differences by route of estrogen or progestin formulation (Model 2, Table 5). However, in a subanalysis using months of use as the predictor, oral estrogen was protective of HTN (aOR [95% CI 0.55–0.95]).

## Conclusions

Overall, we found a strong association that having a recent prescription for progestin increased the odds of TE nearly threefold among transwomen when adjusting for other hormone prescriptions, race, insurance, HIV status, and BMI (Table 5); this association may be stronger for MPA than progesterone. In transwomen, we found that endogenous levels of testosterone were associated with increased odds of HTN only when the model was adjusted for BMI (aOR 1.16, 95% CI 1.01–1.33, *p*=0.04; Table 2). When transwomen were stratified by age group, the odds of increased HTN and increased testosterone levels approached significance only in the older group of transwomen aged 45–70 (aOR 1.21 [95% CI 1.00–1.46], *p*=0.05). We also found that ever having a progestin prescription as protective for HTN among transwomen, with no apparent difference between progesterone and MPA. Overall, we found no associations between hormone use and HTN for transmen; TE events were rare for transmen. MI was a rare event for both transwomen and transmen in this young cohort.

It is fairly accepted that oral ethinyl estradiol, especially at higher doses, increases risk of TE.^7,25^ In addition, at least one study found that unlike ciswomen, the risk for thrombosis among transwomen was cumulative and did not emerge until several years of exposure.^26^ We did not find any positive association between estrogens, including oral estrogens, and TE in our sample; this suggests that safer regimes and better alternatives may now be the standard of care, as we do not prescribe ethinyl estradiol routinely. There is limited evidence that progestins increase the risk of TE, though data have shown an increased risk for injectable (usually MPA) versus oral progestins.^27–29^ Our results also found progestins, particularly MPA, increased the odds of TE, after controlling for estrogen use. Previous work has demonstrated a differential effect between progesterone and MPA on the estradiol signaling pathway, which may have important clinical implications.^30^ Given the observational nature of this analysis and the small sample size, additional work in this area is urgently needed.

Few studies have looked at the association between blood pressure and HT in transwomen. One review found no association among transwomen between blood pressure and HT,^12^ while another study found that estrogens lowered blood pressure in transwomen, possibly due to biologic effects or from lowered stress as they experience gender affirmation.^31^ A recent prospective observational study from Europe found negligible effects of HT on blood pressure in a cohort of transmen and transwomen after 12 months of HT.^9^ We found that a history of progestin, but not estrogen, prescriptions was protective for HTN, though we also found increased duration of oral estrogen use decreased HTN.

Few studies have looked at actual levels of total testosterone. Studies have found an increase in intermediate CVD risk factors associated with testosterone treatment, but no actual increase in CVD events.^22,23,32^ We found that higher endogenous testosterone concentrations were related to a small increase in HTN among transwomen. We also found differences in the association between testosterone and HTN by recent finasteride use but not recent spironolactone use; these results need further examination as we were limited by small samples. Multiple previous studies have shown no association between hormone treatment and CVD for transmen,^6,12,22,23,25^ consistent with our findings; a recent study did find an increase risk for self-reported CVD among transmen, although this was not attributed specifically to hormone use.^33^

There are important limitations to this study. The analysis was observational in nature and clinicians may have prescribed certain hormones based on their patients' CVD risk, which we are unable to control for; likewise, hormone concentrations were available when tests were ordered by clinicians, and may have been more common when there were clinical concerns. Outcomes were based on ICD10 codes, which may be selectively entered by providers. We are unable to assess hormone use before or outside of clinic records. While the use of actual blood concentrations of estradiol and testosterone as a predictor is relatively novel, there can be measurement error particularly in regards to timing of injectable hormones. For instance, reported hormone concentrations may have been drawn at various times during treatment cycles; some represent steady state levels, while others represent peak or trough levels for patients on injectables. Prescriptions are a rich data source, but do not indicate whether a patient actually took the medication. We were able to estimate duration of use based on prescriptions, but there is measurement error and we cannot assess actual adherence. Additionally, data on surgical history of these patients are lacking, thus endogenous hormone production is not accounted for. This is primarily a young population, with access to gender-affirming care; these results may not be generalizable to all transgender patients and additional work is needed regarding nonbinary/gender nonconforming patients who may use different hormone regimens.

Strengths of this work include the large sample size and longitudinal nature, with detailed EMR data, from a U.S. population. We were able to model both lab values and prescription data as predictors, and to separate recent from historical prescription use; we also had a large enough dataset to look at drug routes and specific formulations, though not for all possible combinations. Furthermore, we were able to look at specific types of CVD, rather than looking at all CVD outcomes together, which may blur the effects of specific hormones. Future work is needed to further examine the possible interactions between HIV status, CVD, and HT.^34^

In conclusion, our work shows no increased risk for HTN among transgender patients using HT. These results suggest further work is needed to understand the associations between progestins, particularly MPA, and TE among transwomen; the finding that progestins and oral estrogen may be protective for HTN is reassuring.

## IRB Approval

This study was reviewed and deemed exempt by the Howard Brown Health Center IRB.

## Abbreviations Used

AA: androgen antagonist
aOR: adjusted odds ratio
BMI: body mass index
CI: confidence interval
CVD: cardiovascular disease
EMR: electronic medical records
HT: hormone therapy
HTN: hypertension
OR: odds ratio
TE: thromboembolism
TM: transmen
TW: transwomen
